# 6-[^**18**^F]Fluoro-L-DOPA: A Well-Established Neurotracer with Expanding Application Spectrum and Strongly Improved Radiosyntheses

**DOI:** 10.1155/2014/674063

**Published:** 2014-05-28

**Authors:** M. Pretze, C. Wängler, B. Wängler

**Affiliations:** ^1^Molecular Imaging and Radiochemistry, Department of Clinical Radiology and Nuclear Medicine, Medical Faculty Mannheim of Heidelberg University, Theodor-Kutzer-Ufer 1-3, 68167 Mannheim, Germany; ^2^Biomedical Chemistry, Department of Clinical Radiology and Nuclear Medicine, Medical Faculty Mannheim of Heidelberg University, 68167 Mannheim, Germany

## Abstract

For many years, the main application of [^18^F]F-DOPA has been the PET imaging of neuropsychiatric diseases, movement disorders, and brain malignancies. Recent findings however point to very favorable results of this tracer for the imaging of other malignant diseases such as neuroendocrine tumors, pheochromocytoma, and pancreatic adenocarcinoma expanding its application spectrum. With the application of this tracer in neuroendocrine tumor imaging, improved radiosyntheses have been developed. Among these, the no-carrier-added nucleophilic introduction of fluorine-18, especially, has gained increasing attention as it gives [^18^F]F-DOPA in higher specific activities and shorter reaction times by less intricate synthesis protocols. The nucleophilic syntheses which were developed recently are able to provide [^18^F]F-DOPA by automated syntheses in very high specific activities, radiochemical yields, and enantiomeric purities. This review summarizes the developments in the field of [^18^F]F-DOPA syntheses using electrophilic synthesis pathways as well as recent developments of nucleophilic syntheses of [^18^F]F-DOPA and compares the different synthesis strategies regarding the accessibility and applicability of the products for human *in vivo* PET tumor imaging.

## 1. Introduction

**Figure 1:**
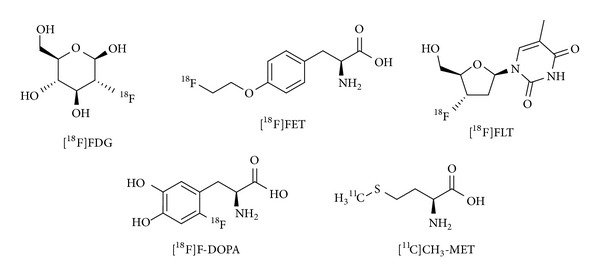
Selected radiotracers applicable in (brain-)tumor imaging.

The ^18^F-radiolabeled nonproteinogenic amino acid 3,4-dihydroxy-6-[^18^F]fluoro-l-phenylalanine ([^18^F]F-DOPA) (Figure 1) has been used for over 30 years to image the presynaptic dopaminergic system in the human brain in order to investigate a number of CNS disorders, in particular schizophrenia [1, 2] and Parkinson's disease with positron emission tomography (PET) [3, 4]. As DOPA is the precursor of the neurotransmitter dopamine, the extent of accumulation of [^18^F]F-DOPA in the brain reflects the functional integrity of the presynaptic dopaminergic synthesis [5] and visualizes the activity of aromatic amino acid decarboxylase (AADC), which converts [^18^F]F-DOPA to ^18^F-dopamine. Likewise, the [^18^F]F-DOPA uptake can also be relevant for determining the effects of treatment of the underlying pathophysiology. For example, its uptake in the striatum is increased during dopamine replacement therapies in Parkinson's disease [6] and modulated by administration of dopamine D_2_ receptor antagonist-based antipsychotic compounds [7, 8]. As a diagnostic tool for the investigation of the neuronal dopaminergic metabolism, a high specific activity (SA) of [^18^F]F-DOPA is not mandatory.

Incidental findings in a patient undergoing a movement disorder diagnosis resulted in a coincidental discovery of a malignant glioma, indicating the potential applicability of [^18^F]F-DOPA also for glioma imaging [9]. In the following, numerous studies were conducted establishing [^18^F]F-DOPA as the main diagnostic tool for brain tumor imaging giving more favorable diagnostic results than [^18^F]FDG [10] (Figure 1) due to a significantly lower background accumulation. Also other alternatives based on amino acids were developed for the imaging of brain malignancies such as [^11^C]methyl-l-methionine ([^11^C]CH_3_-MET) [11–13], 3′-deoxy-3′-l-[^18^F]fluorothymidine ([^18^F]FLT) [14, 15], or [^18^F]fluoroethyl-l-tyrosine ([^18^F]FET) [16–19] (Figure 1) which also exhibit the advantage to show a low physiological accumulation in normal cerebral tissue and inflamed lesions compared to [^18^F]FDG, thus giving more favorable results in brain tumor imaging. Among these tracers used for neurooncologic imaging, [^18^F]F-DOPA shows a high uptake in the malignant tissues, thus allowing a very sensitive tumor detection via PET imaging.

Beyond glioma imaging, recent studies have also shown the increasing importance of [^18^F]F-DOPA for the visualization of various peripheral tumor entities via PET [20] which can be attributed to the upregulation of amino acid transporters in malignant tissues due to an often increased proliferation [21, 22]. [^18^F]F-DOPA, which is transported via the dopamine transporter (DAT) into cells, has thus shown diagnostic advantages in the imaging of high- and low-grade malignancies like neuroendocrine tumors [23–27], pheochromocytoma [28, 29], and pancreatic adenocarcinoma [30–32] regarding diagnostic efficiency and sensitivity. [^18^F]FDG on the contrary is taken up by the glucose transporter not only by malignant tissues but also by inflamed and healthy tissues exhibiting a high glucose metabolism, resulting in low tumor-to-background ratios [10] in CNS malignancies. The proliferation marker [^18^F]FLT which accumulates in malignant tissues due to an enhanced activity of TK1 however often shows relatively low tumor uptakes [15], favoring [^18^F]F-DOPA for the PET imaging of malignancies.

Due to its increasing importance for human tumor imaging, the synthesis of [^18^F]F-DOPA becomes a critical measure regarding its dissemination in clinical routine. Ideally, the radiotracer should be easily accessible in high radiochemical yields (RCYs) and specific activities (SAs) as well as in short synthesis times by an automated process. Furthermore, as it was demonstrated that d-amino acids lack a permeability through the blood-brain barrier, an enantioselective synthesis for [^18^F]F-DOPA is mandatory [33].

The following review outlines the developments in the field of [^18^F]F-DOPA radiosyntheses via electrophilic synthesis routes and the more recent synthesis improvements via nucleophilic syntheses. The main focus of this work is to compare the radiochemical yields (RCYs), radiochemical purities (RCPs), enantiomeric excess (ee), synthesis times, reliability, and a potential for automation of the different radiosynthesis pathways.

## 2. Synthesis Routes for the Production of [**^**18**^**F]F-DOPA

### 2.1. First Attempts to Synthesize [^18^F]F-DOPA

**Figure 2:**
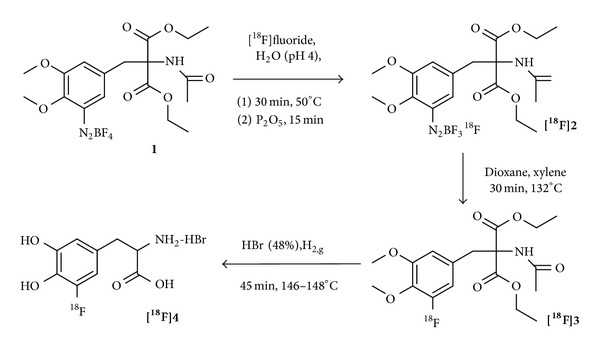
Isotopic exchange reaction pathway for the synthesis of 5-[^18^F]F-DOPA [34].

One of the first fluorine-18-labeled DOPA derivatives was 5-[^18^F]F-DOPA [^**18**^ **F**]**4**, synthesized via isotopic exchange by Firnau et al. in 1973 [34] (Figure 2). In a swimming pool reactor ^6^Li(*n*, ^4^He)^3^H and ^16^O(^3^H, *n*)^18^F nuclear reactions were utilized to produce fluorine-18 in a mixture of Li_2_CO_3_ in H_2_SO_4_ and H_2_O. The resulting [^18^F]fluoride was subsequently distilled twice and the diazonium fluoroborate precursor** 1** was added to this solution. After the isotopic exchange reaction has occurred, the water was removed and the residue was dried over P_2_O_5_. The dried residue [^**18**^ **F**]**2** was redissolved in dioxane, filtered, and heated to 80°C. After adding xylene, the solution was further heated to 132°C for the pyrolysis of the diazonium[^18^F]fluoroborate [^**18**^ **F**]**2** for 30 min. After solvent evaporation, HBr (48%) was added to hydrolyze [^**18**^ **F**]**3** to the final product 5-[^18^F]F-DOPA.

The resulting product [^**18**^ **F**]**4** was obtained in high radiochemical purities of >95% but very low specific activities between 2.2 and 22 kBq/*μ*mol (0.2–2.0 *μ*Ci/mg). Furthermore, the enantiomeric purity of the product was not determined, limiting the applicability of this cumbersome synthesis route.

**Figure 3:**
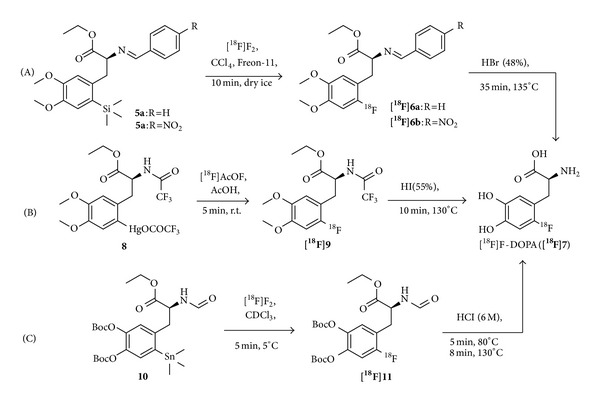
Examples for different demetallation synthesis routes for production of carrier-added [^18^F]F-DOPA ([^**18**^ **F**]**7**) via desilylation (A) [42], demercuration (B) [44], and destannylation (C) [95].

A significant limitation for the use of 5-[^18^F]F-DOPA for* in vivo* imaging purposes is the accelerated* O*-methylation of 5-[^18^F]F-DOPA in contrast to 6-[^18^F]F-DOPA (**[** ^**18**^ **F]7**, Figure 3). This increased* O*-methylation rate is caused by the fluorine atom in position 5 in direct vicinity to the hydroxyl group in position 4 [35] and results in a significantly lower* in vivo* stability of 5-[^18^F]F-DOPA ([^**18**^ **F**]**4**, Figure 2). The same group presented the reaction of [^18^F]F_2_ and l-DOPA in liquid hydrogen fluoride in 1984, yielding a mixture of 2-, 5-, and 6-[^18^F]F-DOPA in low radiochemical yields: 3.7 GBq [^18^F]F_2_ was produced from a Ne-target by a tandem Van de Graaff accelerator to give 111 MBq (3%) 6-[^18^F]F-DOPA, limiting the applicability of this synthesis pathway for a routine production [36].

### 2.2. Electrophilic Syntheses

Twenty years ago, the main route to produce [^18^F]F_2_ for electrophilic fluorination reactions was to utilize the nuclear reaction ^20^Ne(*d*, *α*)^18^F and a F_2_-passivated Ni-target [37]. However, this reaction was limited to facilities with a deuterium accelerator and was thus mostly replaced by the ^18^O(*p*, *n*)^18^F nuclear reaction using a respective ^18^O gas target as this latter method enables the production of higher ^18^F activities [37–39].

To overcome the problem with regioselectivity [40, 41] and the low radiochemical yields obtained by isotopic exchange reactions, radiodemetallation reactions were proposed by several groups. Thus, desilylation [42] and demercuration [43–46] as well as destannylation [47–52] reactions were developed (Figure 3), of which demercuration and destannylation gave the best results and were also adopted to the automated routine production of [^18^F]F-DOPA [53].  Table 1 compares some of the most promising approaches. Multiple purification steps utilizing cartridges, HPLC, and sterile membrane filters were used to remove traces of toxic metal contaminations in the final product solutions to obtain the radiolabeled products in acceptable purities. Nevertheless, using demetallation reactions in a clinical radiotracer production, the final quality control has to include a test for metal contaminants.

Utilizing the carrier-added electrophilic introduction of fluorine-18, the main route to synthesize [^18^F]F-DOPA ([^**18**^ **F**]**7**) is by using commercially available and enantiomerically pure mercury or stannyl precursors such as** 8** or** 10** (Figure 3) in combination with automated synthesis modules [53, 54]. The main advantages are a high enantiomeric purity (ee >99%), short reaction times (about 50 min), and a simplified synthesis setup [54]. However, remaining limitations are the achievable radiochemical yields (25 ± 3%; 0.6–2.6 GBq due to the low production yields of [^18^F]F_2_ from the cyclotron and the substantial loss of at least 50% of activity) and specific activities (4–25 MBq/*μ*mol). As [^18^F]F_2_ can normally be obtained in specific activities of up to 350–600 MBq/*μ*mol [55], the [^18^F]F-DOPA production is not possible in high specific activities by the electrophilic method. Another limitation is the cumbersome transport of gaseous [^18^F]F_2_. Further, the preparation of the precursor compounds is expensive and the radiofluorination of the stannyl precursors gives many side products. In order to obtain [^18^F]F-DOPA in higher SAs and RCYs, it was thus mandatory to develop another synthesis approach. The most promising one is the nucleophilic labeling using no-carrier-added [^18^F]fluoride as it can be obtained in very high specific activities of up to 314–43,000 GBq/*μ*mol [56].

## 3. Nucleophilic Synthesis Strategies for the Production of [**^**18**^**F]F-DOPA

As a tracer for the amino acid metabolism in brain malignancies, a high specific activity is not mandatory for [^18^F]F-DOPA. However, the increasing importance of [^18^F]F-DOPA for peripheral oncologic diagnosis and the need to produce the radiotracer in higher radiochemical yields and specific activities (as too low SAs of [^18^F]F-DOPA were shown to produce pharmacologic effects such as carcinoid crisis by local conversion in tumor tissue of [^18^F]F-DOPA to noradrenaline, induced by the enzymes aromatic acid decarboxylase and dopamine *β*-hydroxylase [57]) resulted in efforts to develop no-carrier-added nucleophilic labeling methods.

### 3.1. Isotopic Exchange

**Figure 4:**
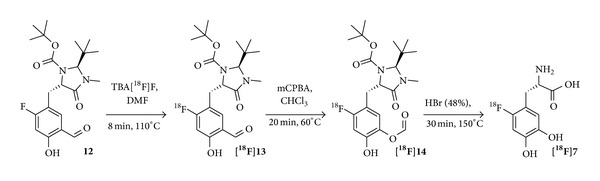
Isotopic exchange reaction for the synthesis of carrier-added [^18^F]F-DOPA [59].

In 2001, Tierling et al. presented the first utilization of an isotopic exchange reaction for the synthesis of [^18^F]F-DOPA [58]. This approach yielded [^18^F]F-DOPA in RCYs of 8–10% (n. d. c.) and an ee of >85% within 70 min. Based on these results, Wagner et al. described the utilization of the isotopic exchange reaction for the radiofluorination of a ^19^F-precursor** 12** with tetrabutylammonium[^18^F]fluoride to produce [^18^F]F-DOPA in high specific activities (Figure 4) [59]. Specific activities in the range of 1.5–2.5 GBq/*μ*mol and RCYs of 22% were calculated to be achievable from a theoretical starting activity of 100 GBq [^18^F]fluoride [60] and ^19^F-precursor amounts of 23 *μ*mol. However, as the reaction was only shown for a starting activity of 370 MBq [^18^F]fluoride and 5.7 *μ*mol ^19^F-precursor and no further isotopic exchange experiments with higher starting activities were demonstrated, the calculated achievable yields of up to 2.5 GBq/*μ*mol remain to be shown.

In 2013, Martin et al. implemented the method of Wagner et al. to a GE TRACERlab MX_FDG_. In preliminary experiments, the automated synthesis of [^18^F]F-DOPA resulted in reproducible RCYs of 10–15% (n. d. c.), RCPs of >95%, and ee of >98% without giving other synthesis details such as reaction times and starting activities [61].

### 3.2. Nucleophilic Syntheses and Aspects of Automation

**Figure 5:**
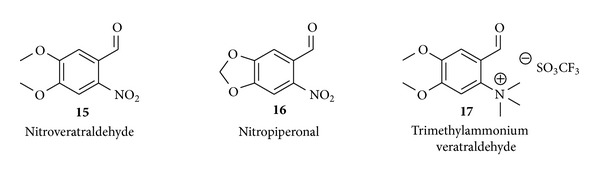
Most common precursors for no-carrier-added nucleophilic radiofluorination reactions producing [^18^F]F-DOPA.

In nucleophilic substitution reactions on aromatic rings using [^18^F]fluoride, the standard leaving groups are mainly nitro- or trimethylammonium moieties (Figure 5) in combination with electron withdrawing groups such as –CO, –CN, and –NO_2_ to enable an efficient reaction. Further, halogen exchange reactions with substituted veratraldehyde (–Cl, –Br, and –F) were evaluated [62]. The first nucleophilic approaches for the synthesis of [^18^F]F-DOPA gave racemates of d- and l-isomers of the tracer which were purified by chiral HPLC resulting in a significant loss of activity [63, 64].

To overcome these problems, new radiosyntheses were developed based on enantiomerically pure chiral precursors or chiral auxiliaries [65–70]. The radiolabeling reactions using these precursors provide the product in moderate to good RCYs accompanied by a high enantiomeric excess of >96%. The most promising approach was published by Lemaire et al. giving [^18^F]F-DOPA in a RCY of 17–29% (d. c.) and a SA of >37 GBq/*μ*mol [66]. In Table 2, selected syntheses using different enantiomerically pure chiral precursors or chiral auxiliaries are compared.

In addition, asymmetric synthesis routes were developed for the radiosynthesis of [^18^F]F-DOPA with higher enantiomeric selectivity and higher RCYs comprising approaches with the precursors depicted in Figure 5 and enantioselective reactions utilizing different chiral phase-transfer catalysts (cPTC). The results from these asymmetric approaches are shown in Table 3.

**Figure 6:**
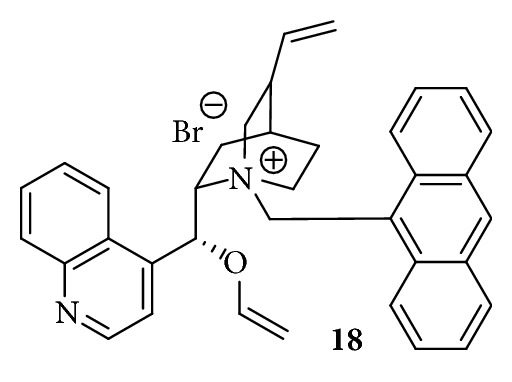
Chiral phase-transfer catalyst* O*-ally-*N*-9-anthracenylmethyl-cinchonidinium bromide** 18**.

A very promising approach for the nucleophilic synthesis of [^18^F]F-DOPA yielding the product in high enantiomeric purities was the utilization of the chiral phase-transfer catalyst* O*-ally-*N*-9-anthracenylmethyl-cinchonidinium bromide (**18**, Figure 6) described by Corey et al. in 1997 [71]. Based on the preliminary results of Lemaire et al. in 1999 [72] and Guillouet et al. in 2001 [73], Zhang et al. adopted the method in 2002 [74] and presented a promising synthesis route utilizing this cPTC** 18** for the enantioselective radiosynthesis of [^18^F]F-DOPA in RCYs of 7–15%, radiochemical purities of >99%, and an ee of 90% within 80–85 min synthesis time. However, special care has to be taken concerning the trimethylammonium veratraldehyde precursor** 17** which exhibits a limited stability upon storage of the precursor for more than six months at 0–4°C resulting in a decreasing RCY for the radiofluorination of** 17** from 40% to <10% [75].

**Figure 7:**
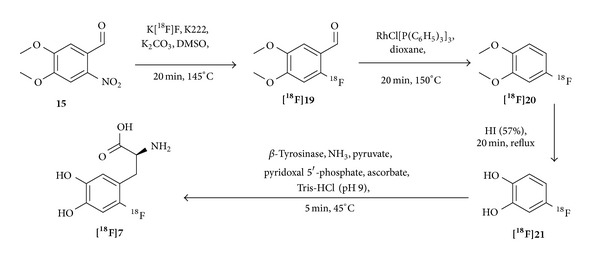
Synthesis pathway for the enzymatic preparation of [^18^F]F-DOPA according to Kaneko et al. [77].

A limitation for this synthesis route is the achievable enantiomeric purities as, according to the European Pharmacopoeia monograph, the limit of the D-enantiomer in the final solution is 2% (ee 96%) [76]. Thus, the synthesis had to be further improved to comply with this limit. A promising approach was presented by Kaneko et al. in 1999 (Figure 7) [77]. The enzymatic reaction step was evaluated carefully and provided a conversion rate of 58% from [^18^F]fluorocatechol ([^**18**^ **F**]**21**) to [^18^F]F-DOPA ([^**18**^ **F**]**7**) under optimized conditions. Despite the efficient enzymatic conversion of [^18^F]F-catechol to the product, the overall RCY of [^18^F]F-DOPA that could be obtained was only 2.0% but resulted in the formation of the product in high SAs of >200 GBq/*μ*mol within 150 min synthesis time. The enantiomeric excess was assumed to be 100% due to the enzymatic character of the reaction although being not confirmed.

The automation of radiotracer syntheses is mandatory for their wide clinical distribution as an automated process gives the product in reproducible quality and limits the radiation exposure to the operating personnel, enabling high starting activities and thus the possibility to synthesize several patient doses in one radiosynthesis.

Therefore, Lemaire et al. optimized the enantioselective reaction using the chiral phase-transfer catalysts** 18** and were able to obtain enantiomeric excesses of about 96% when performing the reaction in toluene at 0°C [78]. However, this reaction setup is difficult to realize in automated processes, due to cooling and heating steps in the same synthesis process. Thus, an optimized synthesis route was developed, preventing the use of diiodosilane. Aldehyde [^**18**^ **F**]**19** and its precursor** 17** (Figure 5) were trapped on a C18 cartridge, the precursor** 17** was removed with water from the solid support, and [^**18**^ **F**]**19** was reduced by aqueous NaBH_4_ and subsequently halogenated by HBr or HI on solid support, resulting in a synthesis setup that could be transferred to an automated synthesis module. Recently, this reaction setup was applied for the radiosynthesis and online conversion from aldehyde [^**18**^ **F**]**19** to different benzyl halides [79].

**Figure 8:**
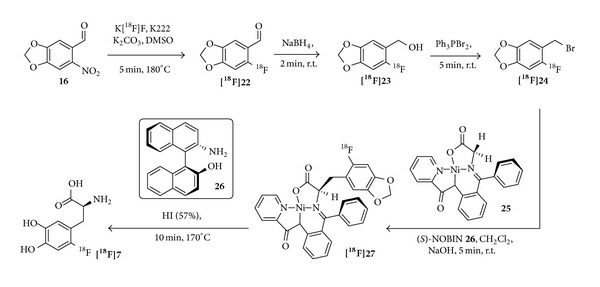
Schematic depiction of the synthesis pathway utilizing NiPBPGly** 25** and (*S*)-NOBIN** 26** as a novel substrate/catalyst pair for the enantioselective radiosynthesis of [^18^F]F-DOPA by Krasikova et al. [80].

Another very promising approach was presented in 2004 by Krasikova et al. [80]. An automated enantioselective radiosynthesis utilizing a novel substrate/catalyst pair, namely, NiPBPGly** 25** and (*S*)-NOBIN** 26** (Figure 8), was developed. In the key alkylation step, the electrophilic bromide [^**18**^ **F**]**2** reacts with the nickel complex** 25** in the presence of (*S*)-NOBIN to form the (*S*)-complex [^**18**^ **F**]**27**. This enantioselective reaction step was accomplished at room temperature, which is favorable in terms of automation. Subsequently, the alkylation was quenched by HI or acetic acid before the solvent was removed in order to prevent racemization of the (*S*)-complex. Different purification steps were optimized to remove any potentially toxic substances present during the synthesis (Ni, Br, P, or B) which was confirmed by ICP-MS analysis of the final product. Using this method, [^18^F]F-DOPA was synthesized in an ee of 96% and RCYs of 16 ± 5% [80] in a total synthesis time of 110–120 min. Although this approach seems to be promising, it has not found a widespread application so far which may be due to the laborious synthesis of the catalyst pair [81, 82] and the challenging purification procedures required for the synthesis which include self-made columns/cartridges in order to remove intermediate reagents and side products.

**Figure 9:**
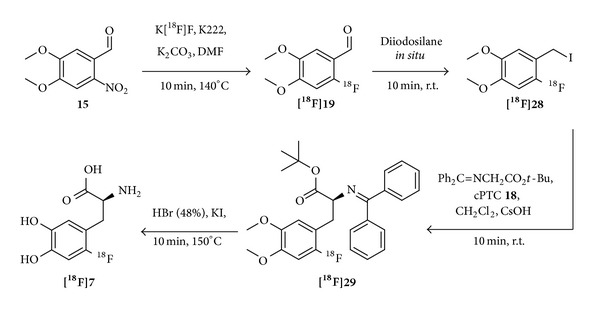
Automated radiosynthesis procedure for [^18^F]F-DOPA using the chiral phase transfer catalyst** 18** [83].

The optimization efforts towards an automation for the routine production of [^18^F]F-DOPA finally resulted in promising synthesis approaches recently. In 2009, Shen et al. presented a method for the fully automated synthesis for [^18^F]F-DOPA [83] utilizing the cPTC** 18** which can be performed at ambient temperature (Figure 9), combining the methods described by Zhang et al. [74] and Lemaire et al. [78]. By optimization of the amounts of reagents during the alkylation process, they were able to obtain [^18^F]F-DOPA in RCYs of 20 ± 4%, SAs of ~50 GBq/*μ*mol, and ee of ≥95% within 120 min synthesis time. In order to obtain higher RCYs, it is important to radiolabel the nitro precursor** 15** in DMF instead of DMSO due to oxidation processes of the aldehyde** 15** occurring in DMSO [84, 85]. Furthermore, the utilization of HBr in combination with KI in the deprotection step resulted in higher RCYs compared to HI alone. However, as noncharacterized substances precipitate during the synthesis, a limitation of this method is the cumbersome maintenance of the synthesis module after each synthesis. To overcome this obstacle, the use of a cassette module would be favorable as this approach would not require the elaborate purification of the module after each use.

**Figure 10:**
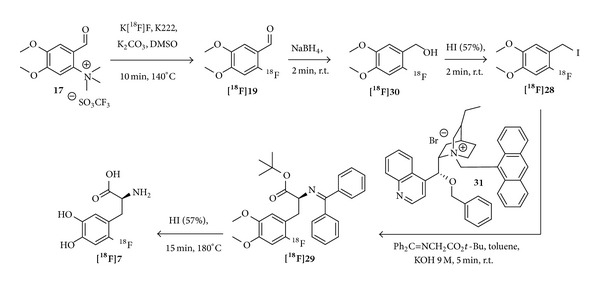
Schematic depiction of the automated synthesis pathway using the chiral phase-transfer catalyst** 31** [86].

Libert and coworkers investigated different cPTC regarding their potential to produce [^18^F]F-DOPA in the highest enantiomeric excesses and high enantiomeric purities of >97% could be obtained under mild reaction conditions within short reaction times [86]. Together with the use of a structurally optimized chiral phase-transfer catalyst (**31**) [71, 87] (Figure 10), a much simplified synthesis setup for automation was enabled. With this optimization, the group of Libert and Lemaire was able to establish a fast automated synthesis and reported product amounts of >45 GBq obtained in RCYs of 24% (n. d. c.) and specific activities of >750 GBq/*μ*mol [86] within 63 minutes (Figure 10). Furthermore, utilizing cPTC** 31** as the catalyst, an ee of >97% could be achieved.

### 3.3. Miscellaneous

In this chapter, some unconventional approaches for the production of [^18^F]F-DOPA are described.

In 2008, Forsback et al. presented an electrophilic labeling approach for the production of [^18^F]F-DOPA in RCYs of 6.4 ± 1.7% (d. c.) and SAs of 3.7 ± 0.9 GBq/*μ*mol [88]. The key step was the synthesis of [^18^F]F_2_ in an electrical discharge chamber by a ^18^F/^19^F-exchange reaction. The ^18^F-source was [^18^F]fluoromethane, which was mixed with a low amount (1 *μ*mol) of carrier fluorine in neon (Ne/0.5% F_2_) inside the discharge chamber. [^18^F]Fluoromethane was produced from methyliodide by a nucleophilic substitution reaction with K[^18^F]F/K222 in acetonitrile. Deuterated solvents for the synthesis of [^18^F]F-DOPA like CDCl_3_, CD_2_Cl_2_, and C_3_D_6_O were also investigated providing significantly higher yields than Freon-11 [89].

**Figure 11:**
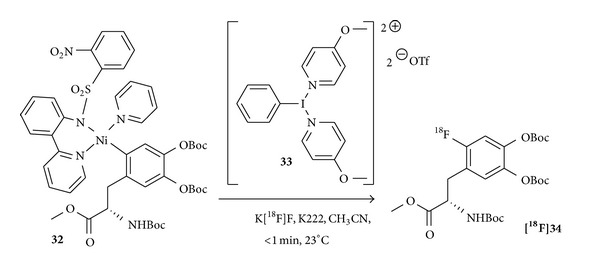
Schematic depiction of an oxidative fluorination approach using the nickel complex** 32** and a hypervalent iodine oxidant** 33** giving the Boc-protected [^18^F]F-DOPA-analogue** [** ^**18**^ **F]34** [90].

In 2012, Lee et al. presented a very fast oxidative fluorination approach for ^18^F-aryl compounds utilizing a nickel-complex** 32** and [^18^F]fluoride (Figure 11). Nickel complex** 32** (1 mg), a hypervalent iodine oxidant** 33** (1 eq.), an aqueous solution of [^18^F]fluoride (2−5 *μ*L, 3.7−18.5 MBq), and K222 (2.0 mg) in acetonitrile (200−500 *μ*L) at 23°C yielded a Boc-protected [^18^F]F-DOPA-analogue [^**18**^ **F**]**34** in RCYs of 15 ± 7% (n. d. c.) in less than 1 minute [90]. This might be also a useful approach for a very fast synthesis of [^18^F]F-DOPA.

In 2013, Stenhagen et al. presented an Ag-mediated electrophilic [^18^F]fluorination of an enantiomerically pure precursor. The protected arylboronic ester was transformed to a 6-Ag-DOPA derivative with silver triflate. Next, [^18^F]selectfluor bis(triflate) in acetone-d_6_ was added. [^18^F]F-DOPA was obtained after 20 min reaction at ambient temperature and 5 min deprotection in RCYs of 19 ± 12% and SAs of 2.6 ± 0.3 GBq/*μ*mol [91]. These results are comparable with the best known electrophilic approaches and could also serve for an automated synthesis.

In summary, radiosynthesis procedures for [^18^F]F-DOPA were developed which can give the radiotracer in high RCYs, SAs, and enantiomeric excesses in short reaction times. Future efforts to even further improve these results could include the utilization of nonoxidizing solvents and microwave conditions in order to achieve even higher [^18^F]fluoride incorporation rates. Up to now, automated systems based on the radiochemistry described by, for example, Wagner et al. [59], Martin et al. [61], and Libert et al. [86] are commercially available.

## 4. Conclusion

In over 30 years, the radiosynthesis of [^18^F]F-DOPA was performed via electrophilic and isotopic exchange routes, when the tracer was mainly applied for the* in vivo* PET imaging of central motor disorders and metabolism imaging purposes. However, the main production route with [^18^F]F_2_ and commercially available stannyl precursors provides [^18^F]F-DOPA in relatively low RCYs and SAs, limiting the use of this promising radiotracer to the imaging of neuronal function and brain malignancies, which is still its main application.

With the discovery of the potential of [^18^F]F-DOPA as radiotracer for the imaging of peripheral malignancies such as neuroendocrine tumors, new radiosynthesis approaches based on nucleophilic substitution reactions were developed, yielding [^18^F]F-DOPA in higher RCYs and SAs as well as shorter synthesis times. Here, two main approaches were followed: one comprises the introduction of nucleophilic [^18^F]fluoride into complex chiral precursors, followed by deprotection and purification, and the other approach starts with introduction of [^18^F]fluoride into simple precursors followed by the utilization of chiral phase-transfer catalysts for an enantioselective synthesis of the product. These processes can also be transferred to automated synthesis modules allowing for a broader dissemination of this favorable radiotracer extending the palette of radiotracers towards a patient-individualized precision medicine.

## Figures and Tables

**Table 1:** Selected synthesis details from electrophilic fluorination reactions for the synthesis of [^18^F]F-DOPA.

Radiolabeling method	Time [min]	RCY [%]^a^	Impurities in product	SA [MBq/*μ*mol]	ee [%]	Citation
Desilylation	60	8^b^	n. d.	25.2	100	Diksic and Farrokhzad '85 [42]
L-DOPA + BF_3_	120	18	n. d.	n. d.	100	Chirakal et al. '86 [92]
Demercuration	65	12	<10 ppb Hg	n. d.	97	Adam and Jivan '88 [43]
Demercuration	50	11	<20 ppb Hg	2.6	>99	Luxen et al. '90 [44]
Destannylation	60	25	<15 ppb Sn	n. d.	>99	Namavari et al. '92 [47]
*O*-Pivaloyl ester of L-DOPA	60	17 ± 1.9	n. d.	17 ± 2.5	100	Ishiwata et al. '93 [93]
Demercuration	45–50	14^b^	<0.05 *μ*g/mL Hg	17–19	>98	Chaly et al. '93 [94]
Destannylation	45–50	26	1.5–2.5 ppm Sn	4.4	>99	Dollé et al. '98 [48]
Destannylation	50	25 ± 3	<1 *μ*g/mL CDCl_3_	30 ± 2	96 ± 1	Füchtner et al. '08 [95]

^a^Unless otherwise stated, RCYs are given decay corrected (d. c.) and ^b^nondecay corrected (n. d. c.).

**Table 2:** Selected synthesis parameters using chiral auxiliaries or precursors.

Precursor	Time [min]	RCY [%] ^18^F-label.	RCY [%] overall^a^	SA [GBq/*μ*mol]	ee [%]	Citation
**16 **	100–110	51	12^b^	n. d.	n. d.	Ding et al. '90 [63]
**15** or **16**	120	n. d.	5–10	n. d.	50 (rac.)	Lemaire et al. '91 [65]
**15**	110	n. d.	5–10^b^	n. d.	83–96	Lemaire et al. '93 [67]
**15** or **16**	120	20–35; ~50	3–5^b^	n. d.	>99	Reddy et al. '93 [68]
**15**	90	45 ± 5	17–29	>37	>96	Lemaire et al. '94 [66]
**15** or **16**	125	n. d.	4-5^b^	>74	98	Horti et al. '95 [69]
**15**	85	~50	6–13^b^	>7.4	98	Najafi '95 [70]

^a^Unless otherwise stated, RCYs are given decay corrected (d. c.) and ^b^nondecay corrected (n. d. c.).

**Table 3:** Selected synthesis parameters utilizing chiral phase-transfer catalysts (cPTC) or asymmetric synthesis routes.

Precursor	Method	Time [min]	RCY [%] ^18^F-label.	RCY [%] overall^a^	SA [GBq/*μ*mol]	ee [%]	Citation
**15**	Enzymatic	150	27	2	>200	>99	Kaneko et al. '99 [77]
**17**	cPTC **18** ^c^	110	n. d.	10–15^b^	74–185	95	Guillouet et al. '01 [73]
**17**	cPTC **18**	80–85	10–40	7–15	n. d.	90	Zhang et al. '02 [74]
**16**	Catalyst **25** ^d^	120	53	16 ± 5	n. d.	96	Krasikova et al. '04 [80]
**17**	cPTC **18**	100	40–50	25–30	n. d.	96	Lemaire et al. '04 [78]
**15**	cPTC **18**	120	71	20 ± 4	>50	≥95	Shen et al. '09 [83]
**17**	cPTC **31** ^e^	63	50	36 ± 3	>750	>97	Libert et al. '13 [86]

^a^Unless otherwise stated, RCYs are given decay corrected (d. c.) and ^b^nondecay corrected (n. d. c.); ^c^see Figure 6; ^d^see Figure 8; ^e^see Figure 10.

## References

[1] Howes OD, Montgomery AJ, Asselin M-C, Murray RM, Grasby PM, Mcguire PK (2007). Molecular imaging studies of the striatal dopaminergic system in psychosis and predictions for the prodromal phase of psychosis. *British Journal of Psychiatry*.

[2] Bose SK, Turkheimer FE, Howes OD (2008). Classification of schizophrenic patients and healthy controls using [^18^F] fluorodopa PET imaging. *Schizophrenia Research*.

[3] Brooks DJ (2003). PET studies on the function of dopamine in health and Parkinson’s disease. *Annals of the New York Academy of Sciences*.

[4] Brooks DJ, Frey KA, Marek KL (2003). Assessment of neuroimaging techniques as biomarkers of the progression of Parkinson’s disease. *Experimental Neurology*.

[5] Cumming P, Deep P, Rousset O, Evans A, Gjedde A (1997). On the rate of decarboxylation of Dopa to Dopamine in living mammalian brain. *Annals of the New York Academy of Sciences*.

[6] Nakamura T, Dhawan V, Chaly T (2001). Blinded positron emission tomography study of dopamine cell implantation for Parkinson’s disease. *Annals of Neurology*.

[7] Tedroff J, Torstenson R, Hartvig P (1998). Effect*s* of the substituted (*S*)-3-phenylpiperidine (−)-OSU6162 on PET measurements in subhuman primates: evidence for tone-dependent normalization of striatal dopaminergic activity. *Synapse*.

[8] Torstenson R, Hartvig P, Långström B, Bastami S, Antoni G, Tedroff J (1998). Effect of apomorphine infusion on dopamine synthesis rate relates to dopaminergic tone. *Neuropharmacology*.

[9] Heiss WD, Wienhard K, Wagner R (1996). F-Dopa as an amino acid tracer to detect brain tumors. *Journal of Nuclear Medicine*.

[10] Chen W, Silverman DHS, Delaloye S (2006). ^18^F-FDOPA PET imaging of brain tumors: comparison study with ^18^F-FDG PET and evaluation of diagnostic accuracy. *Journal of Nuclear Medicine*.

[11] Oka S, Okudaira H, Ono M (2013). Differences in transport mechanisms of *trans*-1-amino-3-[^18^F]fluorocyclobutanecarboxylic acid in inflammation, prostate cancer, and glioma cells: comparison with L-[Methyl-^11^C]methionine and 2-deoxy-2-[^18^F]fluoro-D-glucose. *Molecular Imaging and Biology*.

[12] Okochi Y, Nihashi T, Fujii M (2013). Clinical use of ^11^C-methionine and ^18^F-FDG-PET for germinoma in central nervous system. *Annals of Nuclear Medicine*.

[13] Takenaka S, Asano Y, Shinoda J (2014). Comparison of ^11^C-methionine, ^11^C-chlorine, and ^18^F-fluorodeoxyglucose-PET for distinguishing glioma recurrence from radiation necrosis. *Neurologia Medico-Chirurgica*.

[14] Chen W, Cloughesy T, Kamdar N (2005). Imaging proliferation in brain tumors with ^18^F-FLT PET: comparison with ^18^F-FDG. *Journal of Nuclear Medicine*.

[15] Been LB, Suurmeijer AJH, Cobben DCP, Jager PL, Hoekstra HJ, Elsinga PH (2004). [^18^F]FLT-PET in oncology: current status and opportunities. *European Journal of Nuclear Medicine and Molecular Imaging*.

[16] Galldiks N, Stoffels G, Ruge MI (2013). Role of *O*-(2-^18^F-fluoroethyl)-L-tyrosine PET as a diagnostic tool for detection of malignant progression in patients with low-grade glioma. *Journal of Nuclear Medicine*.

[17] Piroth MD, Prasath J, Willuweit A (2013). Uptake of *O*-(2-[^18^F]fluoroethyl)-L-tyrosine in reactive astrocytosis in the vicinity of cerebral gliomas. *Nuclear Medicine and Biology*.

[18] Sai KK, Huang C, Yuan L (2013). ^18^F-AFETP, ^18^F-FET, and ^18^F-FDG imaging of mouse DBT gliomas. *Journal of Nuclear Medicine*.

[19] Zhang K, Langen KJ, Neuner I (2014). Relationship of regional cerebral blood flow and kinetic behaviour of *O*-(2-^18^F-fluoroethyl)-L-tyrosine uptake in cerebral gliomas. *Nuclear Medicine Communications*.

[20] Seibyl JP, Chen W, Silverman DHS (2007). 3,4-dihydroxy-6-[^18^F]-fluoro-L-phenylalanine positron emission tomography in patients with central motor disorders and in evaluation of brain and other tumors. *Seminars in Nuclear Medicine*.

[21] Isselbacher KJ (1972). Sugar and amino acid transport by cells in culture: differences between normal and malignant cells. *The New England Journal of Medicine*.

[22] Busch H, Davis JR, Honig GR, Anderson DC, Nair PV, Nyhan WL (1959). The uptake of a variety of amino acids into nuclear proteins of tumors. *Cancer Research*.

[23] Neels OC, Koopmans KP, Jager PL (2008). Manipulation of [^11^C]-5-hydroxytryptophan and 6-[^18^F]fluoro-3,4-dihydroxy-L-phenylalanine accumulation in neuroendocrine tumor cells. *Cancer Research*.

[24] Minn H, Kauhanen S, Seppänen M, Nuutila P (2009). ^18^F-FDOPA: a multiple-target molecule. *Journal of Nuclear Medicine*.

[25] Jager PL, Chirakal R, Marriott CJ, Brouwers AH, Koopmans KP, Gulenchyn KY (2008). 6-L-^18^F-fluorodihydroxyphenylalanine pet in neuroendocrine tumors: basic aspects and emerging clinical applications. *Journal of Nuclear Medicine*.

[26] Balogova S, Talbot J-N, Nataf V (2013). ^18^F-Fluorodihydroxyphenylalanine vs other radiopharmaceuticals for imaging neuroendocrine tumours according to their type. *European Journal of Nuclear Medicine and Molecular Imaging*.

[27] Chondrogiannis S, Grassetto G, Marzola MC (2012). ^18^F-DOPA PET/CT biodistribution consideration in 107 consecutive patients with neuroendocrine tumours. *Nuclear Medicine Communications*.

[28] Martiniova L, Cleary S, Lai EW (2012). Usefulness of [^18^F]-DA and [^18^F]-DOPA for PET imaging in a mouse model of pheochromocytoma. *Nuclear Medicine and Biology*.

[29] Rischke HC, Benz MR, Wild D (2012). Correlation of the genotype of paragangliomas and pheochromocytomas with their metabolic phenotype on 3,4-dihydroxy-6-^18^F-fluoro-L- phenylalanin PET. *Journal of Nuclear Medicine*.

[30] Tuomela J, Forsback S, Haavisto L (2013). Enzyme inhibition of dopamine metabolism alters 6-[^18^F]FDOPA uptake in orthotopic pancreatic adenocarcinoma. *EJNMMI Research*.

[31] Jadvar H (2012). Hepatocellular carcinoma and gastroenteropancreatic neuroendocrine tumors: potential role of other positron emission tomography radiotracers. *Seminars in Nuclear Medicine*.

[32] Koopmans KP, Neels OC, Kema IP (2008). Improved staging of patients with carcinoid and islet cell tumors with ^18^F-dihydroxy-phenyl-alanine and ^11^C-5-hydroxy-tryptophan positron emission tomography. *Journal of Clinical Oncology*.

[33] Oldendorf WH (1973). Stereospecificity of blood-brain barrier permeability to amino acids. *The American Journal of Physiology*.

[34] Firnau G, Nahmias C, Garnett S (1973). The preparation of [^18^F]5-fluoro-DOPA with reactor-produced fluorine-^18^. *The International Journal of Applied Radiation and Isotopes*.

[35] Firnau G, Sood S, Pantel R, Garnett S (1981). Phenol ionization in dopa determines the site of methylation by catechol-*O*-methyltransferase. *Molecular Pharmacology*.

[36] Firnau G, Chirakal R, Garnett ES (1984). Aromatic radiofluorination with [^18^F]fluorine gas: 6-[^18^F]fluoro-L-dopa. *Journal of Nuclear Medicine*.

[37] Nickles RJ, Daube ME, Ruth TJ (1984). An ^18^O_2_ target for the production of [^18^F]F_2_. *International Journal of Applied Radiation and Isotopes*.

[38] Roberts AD, Oakes TR, Nickles RJ (1995). Development of an improved target for [^18^F]F_2_ production. *Applied Radiation and Isotopes*.

[39] Hess E, Sichler S, Kluge A, Coenen HH (2002). Synthesis of 2-[^18^F]fluoro-L-tyrosine via regiospecific fluoro-de-stannylation. *Applied Radiation and Isotopes*.

[40] Hatano K, Ishiwata K, Yanagisawa T (1996). Co production of 2, 6-[^18^F]difluoroDOPA during electrophilic synthesis of 6-[^18^F]fluoro-L-DOPA. *Nuclear Medicine and Biology*.

[41] Coenen HH, Franken K, Kling P, Stöcklin G (1988). Direct electrophilic radiofluorination of phenylalanine, tyrosine and dopa. *Applied Radiation and Isotopes*.

[42] Diksic M, Farrokhzad S (1985). New synthesis of fluorine-18-labeled 6-fluoro-L-dopa by cleaving the carbon-silicon bond with fluorine. *Journal of Nuclear Medicine*.

[43] Adam MJ, Jivan S (1988). Synthesis and purification of L-6-[^18^F]fluorodopa. *Applied Radiation and Isotopes*.

[44] Luxen A, Perlmutter M, Bida GT (1990). Remote, semiautomated production of 6-[^18^F]fluoro-L-dopa for human studies with PET. *Applied Radiation and Isotopes*.

[45] Bishop A, Satyamurthy N, Bida G, Barrio JR (1996). Chemical reactivity of the ^18^F electrophilic reagents from the ^18^
*O(p, n)*
^18^F gas target systems. *Nuclear Medicine and Biology*.

[46] Chaly T, Bandyopadhyay D, Matacchieri R (1994). A disposable synthetic unit for the preparation of 3-*O*-methyl-6-[^18^F]fluorodopa using a regioselective fluorodemercuration reaction. *Applied Radiation and Isotopes*.

[47] Namavari M, Bishop A, Satyamurthy N, Bida G, Barrio JR (1992). Regioselective radiofluorodestannylation with [^18^F]F_2_ and [^18^F]CH_3_COOF: a high yield synthesis of 6-[^18^F]fluoro-L-dopa. *Applied Radiation and Isotopes*.

[48] Dollé F, Demphel S, Hinnen F, Fournier D, Vaufrey F, Crouzel C (1998). 6-[^18^F]fluoro-L-DOPA by radiofluorodestannylation: a short and simple synthesis of a new labelling precursor. *Journal of Labelled Compounds and Radiopharmaceuticals*.

[49] Füchtner F, Angelberger P, Kvaternik H, Hammerschmidt F, Simovc BP, Steinbach J (2002). Aspects of 6-[^18^F]fluoro-L-DOPA preparation: precursor synthesis, preparative HPLC purification and determination of radiochemical purity. *Nuclear Medicine and Biology*.

[50] Füchtner F, Steinbach J (2003). Efficient synthesis of the ^18^F-labelled 3-*O*-methyl-6-[^18^F]fluoro-L-DOPA. *Applied Radiation and Isotopes*.

[51] Chang CW, Wang HE, Lin HM, Chtsai CS, Chen JB, Liu R-S (2000). Robotic synthesis of 6-[^18^F]fluoro-L-dopa. *Nuclear Medicine Communications*.

[52] Adam MJ, Lu J, Jivan S (1994). Stereoselective synthesis of 3-*O*-methyl-6-[^18^F]fluorodopa via fluorodestannylation. *Journal of Labelled Compounds and Radiopharmaceuticals*.

[53] de Vries EFJ, Luurtsema G, Brüssermann M, Elsinga PH, Vaalburg W (1999). Fully automated synthesis module for the high yield one-pot preparation of 6-[^18^F]fluoro-L-DOPA. *Applied Radiation and Isotopes*.

[54] Luxen A, Guillaume M, Melega WP, Pike VW, Solin O, Wagner R (1992). Production of 6-[^18^F]fluoro-L-DOPA and its metabolism in vivo: a critical review. *Nuclear Medicine and Biology*.

[55] Hess E, Blessing G, Coenen HH, Qaim SM (2000). Improved target system for production of high purity [^18^F]fluorine via the ^18^
*O(p,n)*
^18^F reaction. *Applied Radiation and Isotopes*.

[56] Füchtner F, Preusche S, Mäding P, Zessin J, Steinbach J (2008). Factors affecting the specific activity of [^18^F]fluoride from a [^18^O]water target. *Nuklearmedizin*.

[57] Koopmans KP, Brouwers AH, De Hooge MN (2005). Carcinoid crisis after injection of 6-^18^F- fluorodihydroxyphenylalanine in a patient with metastatic carcinoid. *Journal of Nuclear Medicine*.

[58] Tierling T, Hamacher K, Coenen HH (2001). A new nucleophilic asymmetric synthesis of 6-[^18^F]fluoro-dopa. *Journal of Labelled Compounds and Radiopharmaceuticals*.

[59] Wagner FM, Ermert J, Coenen HH (2009). Three-step, “one-pot” radiosynthesis of 6-fluoro-3,4-dihydroxy-l-phenylalanine by isotopic exchange. *Journal of Nuclear Medicine*.

[60] Wagner FM (2007). *Zur Synthese radiofluorierter aromatischer Aminosäuren mittels Isotopenaustausch am Beispiel von 6-[^18^F]Fluor-L-DOPA, " [Ph.D. thesis]*.

[61] Martin R, Baumgart D, Hübner S (2013). Automated nucleophilic one-pot synthesis of ^18^F-L-DOPA with high specific activity using the GE TRACERlab MX*FDG*. *Journal of Labelled Compounds and Radiopharmaceuticals*.

[62] Al-Labadi A, Zeller K-P, Machulla H-J (2006). Synthesis of 6-[^18^F]fluoroveratraldehyde by nucleophilic halogen exchange at electron-rich precursors. *Journal of Radioanalytical and Nuclear Chemistry*.

[63] Ding Y-S, Shiue C-Y, Fowler JS, Wolf AP, Plenevaux A (1990). No-carrier-added (NCA) aryl [^18^F]fluorides via the nucleophilic aromatic substitution of electron-rich aromatic rings. *Journal of Fluorine Chemistry*.

[64] Lemaire C, Guillaume M, Cantineau R, Christiaens L (1990). No-carrier-added regioselective preparation of 6-[^18^F]fluoro-L-dopa. *Journal of Nuclear Medicine*.

[65] Lemaire C, Guillaume M, Cantineau R, Plenevaux A, Christiaens L (1991). An approach to the asymmetric synthesis of L-6-[^18^F]fluorodopa via NCA nucleophilic fluorination. *Applied Radiation and Isotopes*.

[66] Lemaire C, Damhaut P, Plenevaux A, Comar D (1994). Enantioselective synthesis of 6-[fluorine-18]-fluoro-L-dopa from no-carrier-added fluorine-18-fluoride. *Journal of Nuclear Medicine*.

[67] Lemaire C, Plenevaux A, Cantineau R, Christiaens L, Guillaume M, Comar D (1993). Nucleophilic enantioselective synthesis of 6-[^18^F]fluoro-L-dopa via two chiral auxiliaries. *Applied Radiation and Isotopes*.

[68] Reddy GN, Haeberli M, Beer H-F, Schubiger AP (1993). An improved synthesis of no-carrier-added (NCA) 6-[^18^F]fluoro-L-DOPA and its remote routine production for PET investigations of dopaminergic systems. *Applied Radiation and Isotopes*.

[69] Horti A, Redmond DE, Soufer R (1995). No-carrier-added (NCA) synthesis of 6-[^18^F]fluoro-L-DOPA using 3,5,6,7,8,8a-hexahydro-7,7,8a-trimethyl-[6*S*-(6*α*, 8*α*, 8*αβ*)]-6,8-methano-2*H*-1,4-benzoxazin-2-one. *Journal of Labelled Compounds and Radiopharmaceuticals*.

[70] Najafi A (1995). Measures and pitfalls for successful preparation of “no carrier added” asymmetric 6-[^18^F]fluor-L-dopa from ^18^F-fluoride ion. *Nuclear Medicine and Biology*.

[71] Corey EJ, Xu F, Noe MC (1997). A rational approach to catalytic enantioselective enolate alkylation using a structurally rigidified and defined chiral quaternary ammonium salt under phase transfer conditions. *Journal of the American Chemical Society*.

[72] Lemaire C, Guillouet S, Plenevaux A, Brihaye C, Aerts J, Luxen A (1999). The synthesis of 6-[^18^F]fluoro-L-dopa by chiral catalytic phase-transfer alkylation. *Journal of Labelled Compounds and Radiopharmaceuticals*.

[73] Guillouet S, Lemaire C, Bonmarchand G, Zimmer L, le Bars D (2001). Large scale production of 6-[^18^F]fluoro-L-DOPA in a semi-automated system. *Journal of Labelled Compounds and Radiopharmaceuticals*.

[74] Zhang L, Tang G, Yin D, Tang X, Wang Y (2002). Enantioselective synthesis of no-carrier-added (NCA) 6-[^18^F]fluoro-L-DOPA. *Applied Radiation and Isotopes*.

[75] Yin D, Zhang L, Tang G, Tang X, Wang Y (2003). Enantioselective synthesis of no-carrier added (NCA) 6-[^18^F]Fluoro-L-Dopa. *Journal of Radioanalytical and Nuclear Chemistry*.

[76] (2008). Fluorodopa (^18^F) (prepared by electrophilic substitution) injection. *European Pharmacopoeia*.

[77] Kaneko S, Ishiwata K, Hatano K, Omura H, Ito K, Senda M (1999). Enzymatic synthesis of no-carrier-added 6-[^18^F]fluoro-L-dopa with *β*- tyrosinase. *Applied Radiation and Isotopes*.

[78] Lemaire C, Gillet S, Guillouet S, Plenevaux A, Aerts J, Luxen A (2004). Highly enantioselective synthesis of no-carrier-added 6-[^18^F]fluoro-L-dopa by chiral phase-transfer alkylation. *European Journal of Organic Chemistry*.

[79] Lemaire C, Libert L, Plenevaux A, Aerts J, Franci X, Luxen A (2012). Fast and reliable method for the preparation of ortho- and para-[^18^F]fluorobenzyl halide derivatives: key intermediates for the preparation of no-carrier-added PET aromatic radiopharmaceuticals. *Journal of Fluorine Chemistry*.

[80] Krasikova RN, Zaitsev VV, Ametamey SM (2004). Catalytic enantioselective synthesis of ^18^F-fluorinated *α*-amino acids under phase-transfer conditions using (*S*)-NOBIN. *Nuclear Medicine and Biology*.

[81] Belokon YN, Kochetkov KA, Churkina TD (2001). Highly efficient catalytic synthesis of alpha-amino acids under phase-transfer conditions with a novel catalyst/substrate pair. *Angewandte Chemie: International Edition*.

[82] Smrčina M, Poláková J, Vyskočil Š, Kočovský P (1993). Synthesis of enantiomerically pure binaphthyl derivatives. Mechanism of the enantioselective, oxidative coupling of naphthols and designing a catalytic cycle. *Journal of Organic Chemistry*.

[83] Shen B, Ehrlichmann W, Uebele M, Machulla H-J, Reischl G (2009). Automated synthesis of n.c.a. [^18^F]FDOPA via nucleophilic aromatic substitution with [^18^F]fluoride. *Applied Radiation and Isotopes*.

[84] Shen B, Löffler D, Reischl G, Machulla H-J, Zeller K-P (2010). Nucleophilic [^18^F]Fluorination and subsequent decarbonylation of methoxy-substituted nitro- and halogen-benzenes activated by one or two formyl groups. *Journal of Labelled Compounds and Radiopharmaceuticals*.

[85] Shen B, Löffler D, Zeller K-P, Übele M, Reischl G, Machulla H-J (2007). Effect of aldehyde and methoxy substituents on nucleophilic aromatic substitution by [^18^F]fluoride. *Journal of Fluorine Chemistry*.

[86] Libert LC, Franci X, Plenevaux AR (2013). Production at the Curie level of no-carrier-added 6-^18^F-fluoro-L-dopa. *Journal of Nuclear Medicine*.

[87] Jew S-S, Park H-G (2009). Cinchona-based phase-transfer catalysts for asymmetric synthesis. *Chemical Communications*.

[88] Forsback S, Eskola O, Haaparanta M, Bergman J, Solin O (2008). Electrophilic synthesis of 6-[^18^F]fluoro-L-DOPA using post-target produced [^18^F]F*2*. *Radiochimica Acta*.

[89] Forsback S, Eskola O, Bergman J, Haaparanta M, Solin O (2009). Alternative solvents for electrophilic synthesis of 6-[^18^F] fluoro-L-DOPA. *Journal of Labelled Compounds and Radiopharmaceuticals*.

[90] Lee E, Hooker JM, Ritter T (2012). Nickel-mediated oxidative fluorination for PET with aqueous [^18^F] fluoride. *Journal of the American Chemical Society*.

[91] Stenhagen ISR, Kirjavainen AK, Forsback SJ (2013). Fluorination of an arylboronic ester using [^18^F]selectfluor bis(triflate): application to 6-[^18^F]fluoro-l-DOPA. *Chemical Communications*.

[92] Chirakal R, Firnau G, Garnett ES (1986). High yield synthesis of 6-[^18^F]fluoro-L-dopa. *Journal of Nuclear Medicine*.

[93] Ishiwata K, Ishii S, Senda M, Tsuchiya Y, Tomimoto K (1993). Electrophilic synthesis of 6-[^18^F]fluoro-L-DOPA: use of 4-*O*-pivaloyl-L-DOPA as a suitable precursor for routine production. *Applied Radiation and Isotopes*.

[94] Chaly T, Dahl JR, Matacchieri R (1993). Synthesis of 6-[^18^F]fluorodopamine with a synthetic unit made up of primarily sterile disposable components and operated by a master slave manipulator. *Applied Radiation and Isotopes*.

[95] Füchtner F, Zessin J, Mäding P, Wüst F (2008). Aspects of 6-[^18^F]fluoro-L-DOPA preparation. *Nuklearmedizin*.

